# Structural basis for the function of SuhB as a transcription factor in ribosomal RNA synthesis

**DOI:** 10.1093/nar/gkz290

**Published:** 2019-04-25

**Authors:** Yong-Heng Huang, Nelly Said, Bernhard Loll, Markus C Wahl

**Affiliations:** 1 Freie Universität Berlin, Laboratory of Structural Biochemistry, Takustraβe 6, D-14195 Berlin, Germany; 2 Helmholtz-Zentrum Berlin für Materialien und Energie, Macromolecular Crystallography, Albert-Einstein-Straße 15, D-12489 Berlin, Germany

## Abstract

Ribosomal RNA synthesis in *Escherichia coli* involves a transcription complex, in which RNA polymerase is modified by a signal element on the transcript, Nus factors A, B, E and G, ribosomal protein S4 and inositol mono-phosphatase SuhB. This complex is resistant to ρ-dependent termination and facilitates ribosomal RNA folding, maturation and subunit assembly. The functional contributions of SuhB and their structural bases are presently unclear. We show that SuhB directly binds the RNA signal element and the C-terminal AR2 domain of NusA, and we delineate the atomic basis of the latter interaction by macromolecular crystallography. SuhB recruitment to a ribosomal RNA transcription complex depends on the RNA signal element but not on the NusA AR2 domain. SuhB in turn is required for stable integration of the NusB/E dimer into the complex. *In vitro* transcription assays revealed that SuhB is crucial for delaying or suppressing ρ-dependent termination, that SuhB also can reduce intrinsic termination, and that SuhB-AR2 contacts contribute to these effects. Together, our results reveal functions of SuhB during ribosomal RNA synthesis and delineate some of the underlying molecular interactions.

## INTRODUCTION

Transcription in bacteria is terminated predominantly *via* two mechanisms (1). Intrinsic termination depends on a stable RNA hairpin followed by a sequence rich in uridines; the hairpin invades the RNA exit tunnel of RNA polymerase (RNAP), while the U-rich stretch forms a weak DNA:RNA hybrid, facilitating termination. In ρ-dependent termination, the hexameric RNA-dependent NTPase, ρ, engages the nascent transcript at C-rich sequences, so-called ρ-utilization (*rut*) sites, uses its NTP-dependent RNA translocase activity to track down RNAP and, upon encounter, leads to termination. Both modes of termination can be supported or suppressed by transcription factors (2). For example, intrinsic termination can be enhanced by transcription factor N-utilization substance (Nus) A that binds RNAP and stabilizes RNA hairpins in the exit tunnel (3–6). ρ-dependent termination can be increased by NusG that also binds RNAP *via* its N-terminal domain (NTD) and contacts ρ *via* its C-terminal domain (CTD), thereby facilitating clamp-down of ρ on RNA at sub-optimal *rut* sites (7). Conversely, NusG can also counteract both modes of termination by enhancing RNAP processivity (8,9), while NusA can inhibit ρ-dependent termination by competing for *rut* sites (10).

As transcription and translation in bacteria are not segregated into different cellular compartments, translation can initiate on mRNAs that are still being transcribed. Indeed, a ribosome trailing RNAP is important for the efficient expression of protein-coding genes, as it hinders ρ from approaching RNAP and thus insulates RNAP from ρ-dependent termination (11). Lack of this effect underlies the principle of translational polarity (12), in which inhibition of translation of an upstream gene in a multi-cistronic mRNA leads to down-regulation of the downstream genes due to premature ρ-dependent transcription termination.

**Figure 1.**
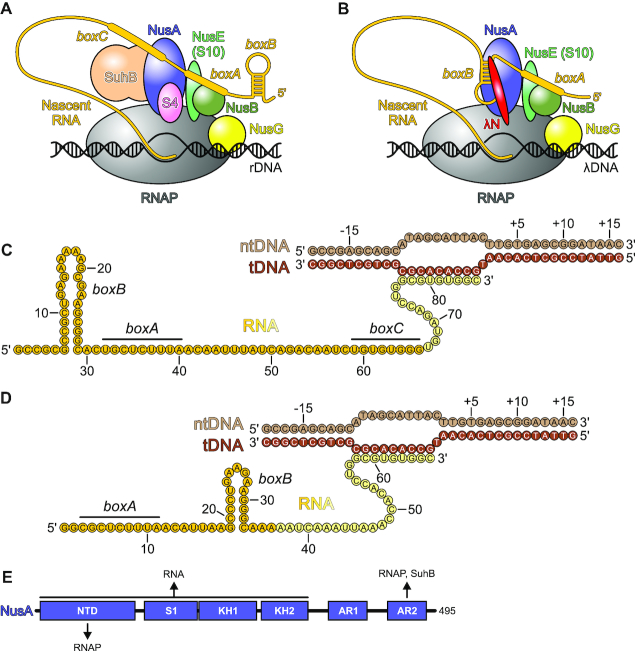
Schemes of processive anti-termination complexes. (**A**) Composition of an *rrn*TAC. (**B**) Composition of a λN-TAC. (**C**) *rrnGnut* RNA used in the present study with *boxB, boxA* and *boxC* highlighted. NtDNA – non-template DNA; tDNA – template DNA. (**D**) Consensus *nut* RNA used in the present study with *boxA* and *boxB* elements highlighted. (**E**) Domain architecture of *E. coli* NusA. Interaction partners in the *rrn*TAC and regions in NusA they bind to are indicated. The newly discovered SuhB-NusA^AR2^ interaction is described in this work.

In bacteria, ρ-dependent termination thus presents a potential obstacle for the efficient synthesis of long, non-coding RNAs, such as ribosomal (r) RNAs, which are not translated. Thus, bacteria might require mechanisms that can prevent such premature termination of rRNA synthesis. Indeed, *Escherichia coli* uses a specialized transcription complex to achieve efficient transcription of rRNA. Initially, Nus factors A, B, E and G (NusE is equivalent to r-protein S10) were recognized as factors participating in this process (13). *In vitro* reconstitution experiments suggested the presence of additional essential components (14), and other r-proteins, in particular S4, were subsequently identified as some of the missing subunits (15). More recently, the inositol mono-phosphatase, SuhB, has been shown to constitute another key player (16). Together, these molecules are thought to assemble a multi-factorial RNA-protein (RNP) complex on the surface of RNAP in response to RNA signal sequences encoded in the ribosomal DNA leader and spacer regions (13,15) (Figure 1A, C). This complex accompanies RNAP during further transcription elongation, forming an rRNA transcription anti-termination complex (*rrn*TAC) that prevents ρ-dependent termination (15) by mechanisms that are presently unclear. The process is thus referred to as processive rRNA (*rrn*) anti-termination.

Processive *rrn* anti-termination is reminiscent of processive anti-termination installed *via* N proteins of lambdoid phages, which is required for the switch from immediate-early to delayed-early gene expression during the lytic life cycle of the phages (17). N-dependent processive anti-termination is also invoked in response to an RNA signal element, the N-utilization (*nut*) site, encoded in leader regions of the phage genomes, which bears a linear element, *boxA*, followed by a hairpin structure, *boxB* (Figure 1B, D). Recent structural analyses by our group have unraveled the structural basis of N-dependent processive anti-termination (18,19). The N protein together with NusA binds the *boxB* element of λ or a consensus *nut* RNA, while the NusB/E dimer recognizes *boxA*. λN strings the Nus factors together, repositioning them on RNAP and presenting a composite NusA-NusE surface that sequesters the CTD of NusG. It thereby prevents NusA-mediated stabilization of RNA hairpins in the exit tunnel and overrides ρ-supporting functions of NusG. Moreover, a C-terminal region of N traverses the RNAP catalytic cavity, stabilizing the enzyme in a processive conformation and counteracting RNA hairpin invasion of the exit tunnel. λN-mediated remodeling of RNAP elements that form part of the RNA exit tunnel and extended guidance of the exiting RNA by repositioned NusA further contribute to hairpin exclusion. λN also cooperates with NusG to stabilize upstream DNA and prevent RNAP backtracking.

The leader and spacer regions in *E. coli* rRNA genes encode RNA signal elements that resemble λ/consensus *nut* sites, bearing *boxB*-like and *boxA*-like elements in the opposite order, followed by an additional linear *boxC* sequence (Figure 1C). As in λN-based anti-termination (20), the *boxA* element serves as a binding site for a hetero-dimer formed by the NusB and NusE subunits (21), and both *boxA* and NusB are required for counteracting ρ in an *in vitro* system (14). Similar signal elements are conserved in the rRNA operons of other bacteria, such as *Mycobacterium tuberculosis*, where *boxA* and *boxC* have been shown to be sequestered in stem-loop structures, and binding of NusA to the *boxC* element is associated with opening of these structures (22). The *boxC* element is also part of a duplex formed with a complementary region in the spacer between the 16S and 23S rRNA portions, which constitutes a processing site for RNase III-mediated excision of pre-16S rRNA (22–24). It has therefore been suggested that NusA may support rRNA maturation by presenting the upstream portion of an RNase III cleavage site to the downstream portion (22).

Presently, SuhB is the least understood subunit of the *rrn*TAC. Based on chromatin immunoprecipitation (ChIP) analyses, it was suggested that SuhB is recruited to the *rrn*TAC in a *boxA*- and NusB-dependent manner (16). *E. coli* SuhB has also been shown to bind RNAP in the form of the holoenzyme (i.e. the α_2_ββ’ω core enzyme in association with a σ initiation factor) (25) and *Pseudomonas aeroguinosa* SuhB has been found associated with RNAP *in vivo* (26). While *E. coli* SuhB possesses inositol-monophosphatase activity, this activity is not required to alleviate effects associated with a *suhB* mutant strain (27). On the other hand, SuhB variants that failed to bind RNAP holoenzyme failed to complement a *suhB* deletion (25), suggesting that SuhB’s transcription-related roles could constitute its main functions in the cell. As SuhB is phylogenetically widely conserved, it is likely that these functions are also widespread in bacteria (16).

Here, we delineated molecular interactions, based on which SuhB participates in rRNA synthesis. We show that SuhB directly binds a C-terminal acidic repeat domain of NusA and contacts at least one other region in NusA as well as the *nut*-like RNA signal element. It thereby facilitates entry of the NusB/E dimer into an *rrn*TAC. Moreover, we determined crystal structures of SuhB alone and in complex with the main SuhB-binding domain of NusA. Transcription assays revealed that SuhB is the critical subunit that elicits delay or suppression of ρ-dependent termination, that an *rrn*TAC comprising SuhB can also suppress intrinsic termination and that these activities depend in part on SuhB interacting with the NusA C-terminal acidic repeat. Based on our results, we suggest molecular mechanisms by which SuhB may support anti-termination. Our results also have implications for SuhB and Nus factors promoting rRNA maturation.

## MATERIALS AND METHODS

### Plasmids, DNAs and RNAs

DNA fragments encoding SuhB and S4 were PCR-amplified from *E. coli* (DH5α) genomic DNA and cloned into the pETM-11 vector (European Molecular Biology Laboratory) *via Nco*I and *Hind*III restriction sites. A DNA fragment encoding NusA^AR2^ (residues 427–495) was PCR-amplified from a pETM-NusA plasmid and cloned into pETM-11 *via Nco*I and *Hind*III restriction sites. A DNA template for *in vitro* transcription assays was generated by assembly PCR and cloned into pUC18 vector *via Xba*I and *Hind*III restriction sites. All constructs were verified by sequencing (Seqlab). DNAs used for the assembly of transcription complexes were purchased as single-stranded oligonucleotides (Eurofines). RNA constructs were synthesized by *in vitro* transcription by T7 P266L RNA polymerase (28), using PCR products as templates, and purified as described (19).

### Protein production and purification

Full-length SuhB was produced in *E. coli* BL21(DE3)pLysS overnight at 37°C in auto-induction medium (29). Cells were harvested by centrifugation and lysed in lysis buffer (50 mM Tris–HCl, pH 7.5, 500 mM NaCl, 1 mM 2-mercaptoethanol). All subsequent steps were performed at 4°C or on ice. Cleared lysate was incubated with Ni^2+^-NTA agarose beads (Macherey-Nagel), beads were washed with lysis buffer supplemented with 20 mM imidazole. Captured protein was eluted with elution buffer (50 mM Tris–HCl, pH 7.5, 200 mM NaCl, 1 mM 2-mercaptoethanol, 400 mM imidazole), digested with TEV protease overnight to cleave the His_6_-tag and purified to homogeneity by size exclusion chromatography on a Superdex 75 26/60 column (GE Healthcare) in storage buffer (20 mM Tris–HCl, 200 mM NaCl, 1 mM DTT). NusA^AR2^ was purified *via* the same protocol as full-length NusA (19).

Plasmids encoding SuhB, NusA and NusA^AR2^ variants were generated by site-directed mutagenesis, and the proteins were produced and purified by the same protocols as used for the wild type (wt) proteins. Other proteins (RNAP, NusA, NusA^ΔAR2^, NusB/E, NusG, ρ and σ70) were produced and purified as described previously (18,19).

### Analytical size exclusion chromatography

Interactions were tested by analytical size exclusion chromatography (SEC). Before loading on a Superdex 200 Increase 3.2/300 column (GE Healthcare), near-stoichiometric amounts of proteins and/or nucleic acids (20 μM final concentration for the largest component and 25 μM final concentrations for all smaller components) were mixed in running buffer (20 mM HEPES–NaOH, pH 7.5, 50 mM NaCl, 1 mM DTT) and incubated at room temperature for 15 min. SEC was conducted with a flow rate of 40 μl/min and 50 μl (40 μl for runs with RNAP) fractions were collected. Fractions were analyzed on 15% SDS-PAGE gels (11–16.5% gradient SDS-PAGE gels for runs with RNAP) and 15% 8 M urea–PAGE gels for proteins and nucleic acids, respectively.

### Surface plasmon resonance assays

Surface plasmon resonance (SPR) experiments were carried out on Biacore T20 (GE Healthcare) using Sensor Chip NTA (GE Healthcare) at 20°C. The Sensor Chip NTA was loaded with Ni^2+^ by incubating in 0.5 mM NiCl_2_. Subsequently, 3 mM EDTA, pH 8.3, was flowed across the chip to remove unbound Ni^2+^. After equilibrating with SPR buffer (20 mM HEPES–NaOH, pH 7.5, 50 mM NaCl, 1 mM DTT), N-terminally His_6_-tagged NusA^AR2^ was immobilized on the chip, excess protein was washed away with 45 μl of SPR buffer (30 μl/min, 90 s). SPR experiments were carried out according to the single cycle-kinetic method, using SuhB variants in SPR buffer at 25, 50, 100, 150 and 200 nM. The same experiments were carried out in parallel in the control channel without prior Ni^2+^ loading. Responses of the control channel were subtracted from the responses in the experimental channel and corrected data were analyzed with Biacore T20 software (GE Healthcare).

### Size exclusion chromatography/multi-angle light scattering

Size exclusion chromatography/multi-angle light scattering analyses were performed on an HPLC system (Agilent) coupled to a mini DAWN TREOS multi-angle light scattering and RefractoMax 520 refractive index detectors (Wyatt Technology). 60 μl (15 nmol) of SuhB were passed over a Superdex 200 increase 10/300 column (GE Healthcare) in 20 mM HEPES, pH 7.5, 50 mM NaCl, 0.02% (w/v) NaN_3_ at a flowrate of 0.6 ml/min. Data were analyzed with the ASTRA 6.1 software (Wyatt Technology) using monomeric bovine serum albumin (Sigma-Aldrich) as a reference.

### Double filter-binding assays

*rrnGnut* or *boxBA* RNA oligos (residues 1–66 and 1–40, respectively; Figure 1C) were 5′-end-labeled using [γ-^32^P]ATP and T4 polynucleotide kinase (moloX) and purified using Microspin G25 columns (GE Healthcare). Increasing concentrations of SuhB were mixed with 50 nM labeled RNAs in 20 mM HEPES–NaOH, pH 7.5, 50 mM NaCl, 1mM DTT (20 μl final volume) and incubated at room temperature for 20 min. Incubated samples were pipetted on sandwiched nitrocellulose (Protran 0.2 NC, Amersham; upper membrane) and nylon (Hybond-N+, GE Healthcare; lower membrane) filters using a multi-well filtration manifold (BIO-RAD) as described (20). Membranes were immediately washed with 200 μl 20 mM HEPES–NaOH, pH 7.5, 50 mM NaCl, 1 mM DTT and air-dried. Results were visualized by autoradiography using a Storm PhosphorImager (GE Healthcare) and quantified with Image-Quant software (GE Healthcare). Data were fit according to a one-site specific binding model with Hill slope: (*B* = *B*_max_•*X^h^*/[*K*_d_^*h*^ + *X*^*h*^]; *B* – fraction bound; *B*_max_ – maximum fraction bound; *X* – concentration of SuhB; *h* – Hill slope; *K*_d_ – dissociation constant).

### Transcription assays

For *in vitro* transcription assays, a DNA template containing a *T7A1con* promotor (*T7A1* bearing a consensus –10 element) followed by the anti-termination region from the *E. coli rrnG* operon, *rutA/rutB* ρ entry and *trpt’* ρ termination regions from the *trp* operon (10) and the *tR'* intrinsic terminator (without the endogenous zone of opportunity) from the phage λ genome was designed. Assays were performed in single-round transcription format (30). 100 nM of *E. coli* RNAP core enzyme and σ70 factor, 20 nM template DNA, 10 μM ApU, 2 μM ATP, GTP and CTP and 2 μCi α-[^32^P]CTP were mixed in 10 μl transcription buffer (20 mM Tris-OAc, pH 7.9, 100 mM KOAc, 5 mM Mg(OAc)_2_, 5% (v/v) glycerol, 1 mM DTT) and incubated at 32°C for 10 min to generate initial transcription complexes with an 11-nucleotide labeled RNA. 200 nM NusA, 1 μM NusG, 1 μM NusB/E, 500 nM S4 and/or 500 nM SuhB, as well as 500 nM hexameric ρ where indicated, were then added to the reaction and incubated for 5 min at 32°C. Subsequently, a mixture of all four rNTPs was added to the reaction (final concentrations of 2 mM ATP and CTP, 100 μM GTP and UTP). Samples were taken at defined time points, PCI-extracted, isopropanol-precipitated and analyzed via 6% 8 M urea–PAGE. Bands were visualized on a Storm PhosphorImager and quantified with Image-Quant software. Relative read-through of the *trpt’* region was determined as the percentage of *trpt’* read-through products relative to all products in a lane, with the value for ρ acting on RNAP alone set to 0 and the values for ρ acting on all other complexes scaled accordingly. Relative read-through of the *tR'* intrinsic terminator was determined as the percentage of *tR'* read-through products relative to all products in a lane with the value for RNAP alone set to 0 and the values for all other complexes scaled accordingly.

### Crystallographic procedures

SuhB protein was mixed with NusA^AR2^ in a 1:1.2 molar ratio in 10 mM Tris–HCl, pH 7.5, 50 mM NaCl, 1 mM DTT. The mixture was injected on a Superdex 75 16/60 size exclusion column (GE Healthcare) to obtain homogenous complex. The purified complex was concentrated to 8 mg/ml. Crystallization was conducted by sitting-drop vapor diffusion in 48-well plates. The best crystals grew upon mixing 1.5 μl of complex solution with 1 μl of reservoir solution containing 100 mM Tris–HCl, pH 8.5, 5% (w/v) PEG 8000, 16% (v/v) PEG 300 and 10% (v/v) glycerol. Crystals were soaked in 100 mM Tris–HCl, pH 8.5, 12.5% (w/v) PEG 8000, 40% (v/v) PEG 300, 10% (v/v) glycerol overnight before being fished and flash-cooled in liquid nitrogen. For isolated SuhB protein, 2 μl of a 10 mg/ml protein solution was mixed with 1 μl reservoir solution (100 mM Tris–HCl, pH 8.3, 12% (w/v) PEG 8000). SuhB crystals were flash-cooled in liquid nitrogen after soaking in artificial mother liquor containing 30% (v/v) PEG 300.

Diffraction data were collected on beamline 14.2 at the BESSY II storage ring (Berlin, Germany) at 100 K. All data were processed with XDS (31,32). Structures were solved by molecular replacement, using the structure coordinates of NusA^AR2^ (PDB ID 1WCN) and/or the SuhB^R184A^ variant (PDB ID 2QFL). Structures were refined by alternating rounds of model building in COOT (33) and automated maximum-likelihood restrained refinement in PHENIX (34). Model quality was evaluated with MolProbity (35) and the JCSG validation server (JCSG Quality Control Check v3.1). Structure figures were prepared using PyMOL (36). Data collection and refinement statistics are provided in Table 1.

**Table 1.** X-ray diffraction data collection and structure refinement statistics^a^

Dataset	SuhB-NusA^AR2^	SuhB
**Data collection**		
**PDB ID**	6IB8	6IB7
**Wavelength [Å]**	0.9184	0.9184
**Temperature [K]**	100	100
**Space group**	*P*2_1_2_1_2_1_	*C*2
**Unit cell parameters**		
Axes [Å]	64.27, 95.54, 104.54	90.72, 46.03, 72.91
Angles [°]	90.0, 90.0, 90.0	90.0, 125.4, 90.0
**Resolution** [Å]	50.00–1.65 (1.74–1.65)	50.00–2.25 (2.38–2.25)
**Reflections**		
Unique	78 162 (12 069)	11 994 (1919)
Completeness [%]	99.1 (95.7)	97.2 (96.8)
Redundancy	5.5 (5.1)	3.3 (3.4)
***I*/σ(*I*)**	15.2 (0.9)	10.7 (1.1)
***R*_meas_(*I*)** [%]^b^	7.8 (186.4)	9.2 (145.0)
**CC_1/2_** [%]^c^	99.9 (36.3)	99.9 (58.3)
**Refinement**		
**Resolution** [Å]	30.00–1.65 (1.68–1.65)	36.98–2.25 (2.47–2.25)
Reflections		
Number	77 933 (4649)	11 611 (2886)
Completeness [%]	99.1 (89.9)	97.3 (97.2)
Test set [%]	2.69	5.00
***R*_work_** ^d^	18.2 (37.6)	21.5 (33.1)
***R*_free_** ^e^	21.9 (41.9)	26.1 (40.0)
**Contents of A.U**.^f^		
Non-H atoms	5111	2044
Protein molecules/residues	3/594	1/257
Mg^2+^ ions	2	1
Glycerol molecules	9	2
PEG units	2	0
Water oxygens	267	30
**Mean *B* factors** [Å^2^]		
Wilson	26.3	54.2
Protein	34.6	65.9
Ligands	44.3	64.6
Water oxygens	37.0	60.1
**Ramachandran plot** ^g^		
Favored [%]	98.1	97.7
Outliers [%]	0.0	0.0
**Rmsd** ^h^		
Bond lengths [Å]	0.015	0.005
Bond angles [°]	1.009	0.736

^a^Values in parentheses refer to the highest resolution shells.

^b^ *R*_meas_(*I*) = ∑_h_ [*N*/(*N* – 1)]^1/2^ ∑_*i*_ │*I_i_*_h_ – <*I*_h_>│ / ∑_h_∑_*i*_*I_i_*_h_, in which <*I*_h_> is the mean intensity of symmetry-equivalent reflections *h, I_i_*_h_ is the intensity of a particular observation of h and N is the number of redundant observations of reflection *h*.

^c^CC_1/2_ = (<*I*^2^> – <*I*>^2^) / (<*I*^2^> – <*I*>^2^) + σ^2^_ϵ_, in which σ^2^_ϵ_ is the mean error within a half-dataset (45).

^d^ *R*_work_ = ∑_*h*_ │*F*_o_ – *F*_c_│ / ∑ *F*_o_ (working set, no σ cut-off applied).

^e^ *R*_free_ is the same as *R*_work_, but calculated on the test set of reflections excluded from refinement.

^f^A.U. – asymmetric unit.

^g^Calculated with MolProbity (35).

^h^Rmsd - root-mean-square deviation from target geometry.

### Structure comparisons

Structures were compared by global superposition of complex structures or by superposition of selected subunits in complexes using the Secondary Structure Matching (SSM) algorithm implemented in COOT (33).

## RESULTS

### SuhB binds directly to NusA and the RNA signal element

**Figure 2.**
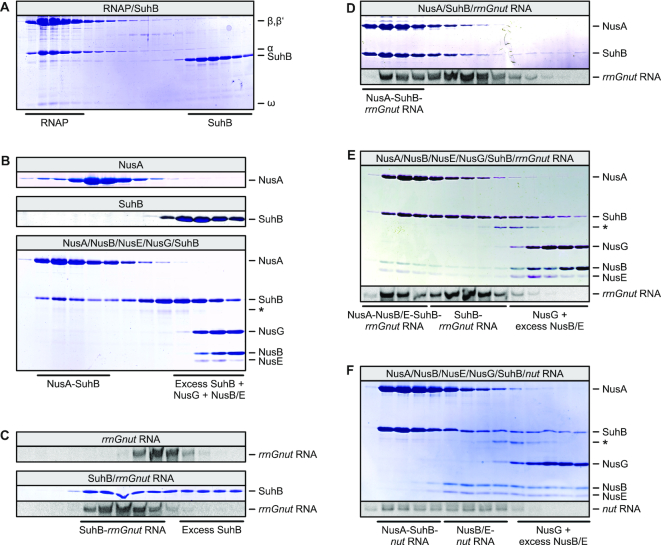
SEC analyses monitoring the interaction of SuhB with other components of the *rrn*TAC. In this and the following figures, protein fractions were analyzed by SDS PAGE, nucleic acid fractions were analyzed by 8 M urea–PAGE; analyzed components or mixtures are identified in the gray box above the gels; fractions corresponding to the elution of specific complexes or isolated components are identified below the gels; bands are identified on the right. (**A**) Lack of binding of SuhB to RNAP core enzyme. (**B**) First and second panel – SEC runs of isolated NusA and SuhB, respectively. Third panel – binding of SuhB to NusA but not to the other Nus factors. In this and the following figures, the same fractions were analyzed in gels showing SEC runs of isolated components and gels showing SEC runs of mixtures. An asterisks in this and several of the following gels denotes a minor contaminant that originated from our NusG preparations and that runs at almost the same position as r-protein S4 in SDS PAGE. (**C**) First panel – SEC run of *rrnGnut* RNA alone. Second panel – binding of SuhB to *rrnGnut* RNA. (**D**) Ternary SuhB-NusA-*rrnGnut* RNA complex formation. The same fractions as in (**C**) are shown. (**E**) Formation of a SuhB-NusA-NusB/E-*rrnGnut* RNA complex. (**F**) Formation of separate SuhB-NusA-*nut* RNA and NusB/E-*nut* RNA complexes with consensus *nut* RNA (labeled ‘*nut* RNA’ for simplicity).

Presently it is unknown which other components of the *rrn*TAC SuhB directly interacts with. To address this question, we conducted analytical size exclusion chromatography (SEC) analyses using recombinantly produced, purified components. R-protein S4 could not be included in these initial experiments, as in the absence of RNAP, DNA and RNA, it tended to aggregate and precipitate with the other factors. Under the chosen conditions, SuhB did not stably interact with RNAP alone (Figure 2A). Likewise, SuhB did not bind the NusB/E dimer or NusG (Figure 2B). However, SuhB co-eluted both with NusA and with *rrnGnut* RNA (residues 1–66; Figure 1C) from the gel filtration column, and these components together eluted earlier than the individual molecules (Figure 2B, C), indicating the formation of stable SuhB-NusA and SuhB-*rrnGnut* RNA complexes under the chosen conditions. SuhB, NusA and *rrnGnut* RNA also formed a ternary complex (Figure 2D).

Upon mixing SuhB with all Nus factors and *rrnGnut* RNA, a complex comprising SuhB, NusA, NusB, NusE and *rrnGnut* RNA eluted from the gel filtration column (Figure 2E). A similar complex could not be assembled when phage λ or consensus *nut* RNAs (residues 1–36; Figure 1D) were used instead (Figure 2F). In the latter case, separate ternary SuhB-NusA-*nut* RNA and NusB-NusE-*nut* RNA complexes formed (Figure 2F).

**Figure 3.**
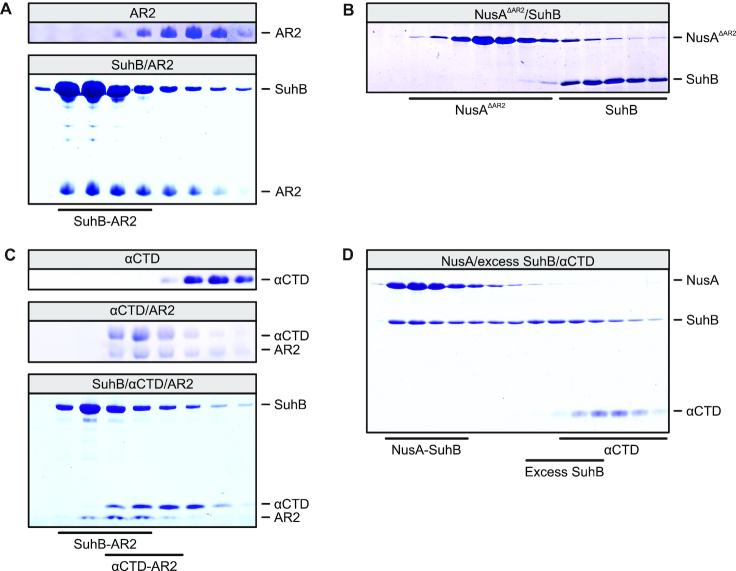
SEC analyses monitoring the interaction of SuhB with NusA variants and RNAP αCTD. (**A**) Binding of NusA^AR2^ to SuhB. First panel – SEC run of isolated NusA^AR2^. Second panel – interaction of SuhB and NusA^AR2^. (**B**) Lack of binding of SuhB to NusA^ΔAR2^. (**C**) SEC analyses demonstrating mutually exclusive binding of SuhB and αCTD to NusA^AR2^. First panel – isolated αCTD. Second panel – binding of αCTD to NusA^AR2^. Third panel – formation of separate SuhB-NusA^AR2^ and αCTD-NusA^AR2^ complexes upon mixing of all three components. (**D**) SEC analysis showing failure of αCTD to join a SuhB-NusA^FL^ complex formed in the presence of excess SuhB.

NusA is a multi-domain protein that encompasses an RNAP-binding NTD, an array of RNA-binding S1 and two hnRNP K homology domains (KH1 and KH2) and two C-terminal acidic repeat domains (AR1 and AR2; Figure 1E). The latter two domains are not universally conserved in NusA orthologs. To test which region of NusA is bound by SuhB, we recombinantly produced various fragments of NusA and repeated the SEC analyses. SuhB bound to a construct comprising the NusA AR2 domain (residues 427–495; NusA^AR2^; Figure 3A), but failed to interact with a NusA variant lacking this domain (residues 1–426; NusA^ΔAR2^; Figure 3B). These results indicate that the AR2 domain of NusA represents the main contact site of the protein to SuhB.

### *RrnGnut* RNA is required for stable integration of SuhB into transcription complexes

**Figure 4.**
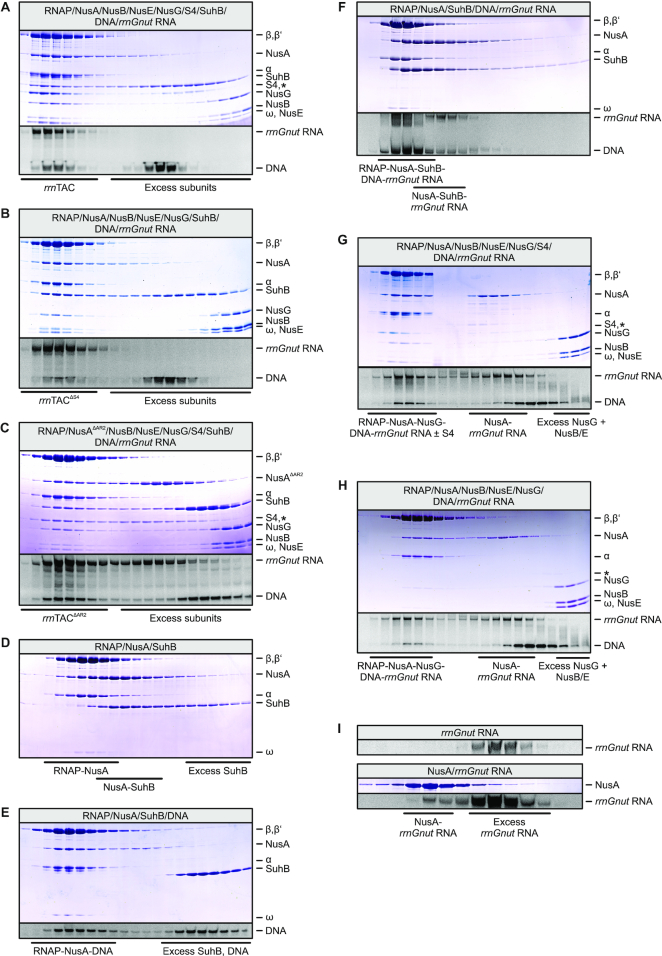
SEC analyses monitoring the formation of transcription complexes. (**A**) Formation of a complete *rrn*TAC. (**B**) SuhB integration does not depend on the presence of r-protein S4. (**C**) SuhB is still efficiently integrated into an *rrn*TAC formed with NusA^ΔAR2^. (**D**–**F**) SuhB fails to join a RNAP-NusA complex (**D**) or a RNAP-NusA-DNA complex (**E**), but associates with RNAP and NusA when a nucleic acid scaffold, in which the RNA bears a *rrnGnut* site, is contained (**F**). (**G, H**) Irrespective of the presence of S4, NusB/E are not integrated into a transcription complex formed with *rrnGnut* RNA in the absence of SuhB. In addition, S4 associates with the complex only partially when SuhB is missing (**G**). Moreover, excess NusA competes with NusB/E for binding to excess *rrnGnut* RNA (**G, H**). (**I**) Interaction of NusA^FL^ with *rrnGnut* RNA in the absence of other factors. First panel – isolated *rrnGnut* RNA (same run shown as in the top panel of Figure 2C). Second panel – mixture of NusA and *rrnGnut* RNA.

Upon mixing RNAP, DNA with an artificial transcription bubble, *rrnGnut* RNA that could pair in the transcription bubble, all Nus factors, S4 and SuhB, a stable *rrn*TAC could be reconstituted (Figure 4A). S4 was not required for formation of an RNAP-DNA-*rrnGnut* RNA-NusA/B/E/G-SuhB complex (Figure 4B). A complex containing all components also still formed when full-length NusA (NusA^FL^) was replaced by a variant lacking the NusA AR2 domain (NusA^ΔAR2^; Figure 4C), indicating that the AR2 domain of NusA is dispensable for integration of SuhB into the *rrn*TAC. In contrast to NusA^AR2^, *rrnGnut* RNA was required for stable integration of SuhB into transcription complexes: SuhB did not join an RNAP–NusA complex (Figure 4D) or a complex containing RNAP, NusA and DNA but lacking *rrnGnut* RNA (Figure 4E); however, it did associate with RNAP–NusA when DNA and *rrnGnut* RNA were also present (Figure 4F).

These observations were unexpected, given that SuhB forms a stable complex with NusA and NusA^AR2^ in isolation (see above). However, the NusA AR2 domain can also interact with the C-terminal domain of the RNAP α subunit (αCTD), as recently visualized in a NusA-modified *his*-operon hairpin-paused elongation complex (6). The NusA^AR2^-αCTD interaction is thought to allow RNA binding by NusA, as RNA binding in isolated NusA is auto-inhibited by AR1–AR2 folding back onto the S1-KH1-KH2 RNA-binding region in a manner mutually exclusive with the NusA^AR2^-αCTD interaction (37,38). We therefore tested whether binding of SuhB and αCTD to NusA^AR2^ is also mutually exclusive. Indeed, while stable SuhB-NusA^AR2^ (Figure 3A) and αCTD-NusA^AR2^ (Figure 3C, middle) complexes formed in analytical SEC upon mixing the respective proteins, a mixture of SuhB, αCTD and NusA^AR2^ gave rise to separate SuhB-NusA^AR2^ and αCTD-NusA^AR2^ complexes eluting from the column (Figure 3C, bottom). In addition, excess SuhB prevented αCTD from associating with NusA (Figure 3D). We therefore suggest that in transcription complexes lacking *rrnGnut* RNA, NusA tends to bind *via* its AR2 domain to the αCTD of RNAP, preventing SuhB-AR2 interactions.

### SuhB facilitates recruitment of NusB/E and S4 to transcription complexes

Interestingly, the NusB/E dimer failed to associate with transcription complexes bearing *rrnGnut* RNA when SuhB was omitted, irrespective of the presence of S4 (Figure 4G, H). In addition, while a NusA-*rrnGnut* RNA complex formed with excess material in these SEC runs, NusB/E did not associate with this NusA-*rrnGnut* RNA complex (Figure 4G,H), suggesting that in the absence of SuhB, NusA competes with NusB/E on *rrnGnut* RNA. Moreover, in the absence of SuhB, S4 only partially associated with *rrnGnut* RNA-containing transcription complexes (Figure 4G).

**Figure 5.**
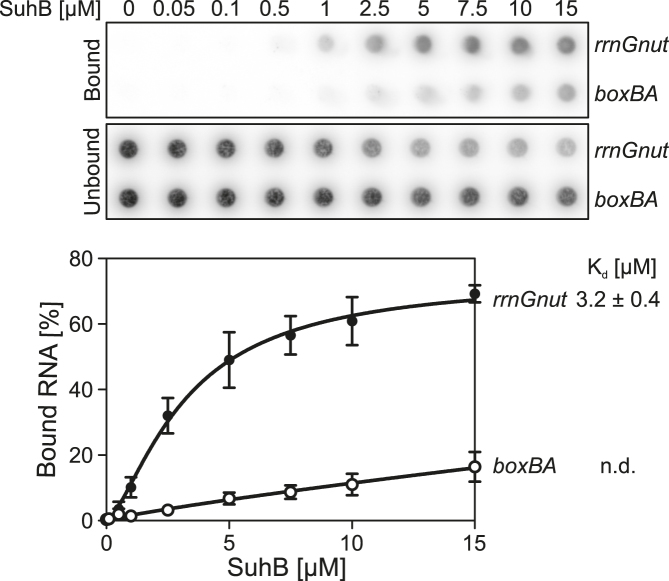
Double nitrocellulose/nylon filter-binding assay, monitoring interaction of SuhB with *rrnGnut* RNA or *boxBA* RNA. Top panel – autoradiograms of the bound (nitrocellulose) and unbound (nylon) fractions of the RNAs at increasing concentrations of SuhB. RNAs are identified on the right. Bottom panel – quantification of the data. Data were fit according to a one-site specific binding model with Hill slope; (*B* = *B*_max_•*X*^*h*^/[*K*_d_^*h*^+*X*^*h*^]; *B* – fraction bound; *B*_max_ – maximum fraction bound; *X* – concentration of SuhB; *h* – Hill slope; *K*_d_ – dissociation constant). The *K*_d_ for the SuhB–*rrnGnut* RNA interaction is indicated on the right; the *K*_d_ for the SuhB–*boxBA* RNA interaction could not be determined (n.d.).

The apparent competitive binding of NusA and NusB/E to *rrnGnut* RNA in the absence of SuhB was surprising, due to the above-mentioned auto-inhibition of isolated NusA with respect to λ/consensus *nut* RNA binding (37,38). However, isolated NusA clearly did bind to *rrnGnut* RNA even in the absence of other factors (Figure 4I), indicating that this RNA might have a high enough affinity to overcome NusA auto-inhibition. In double filter-binding assays, SuhB bound *rrnGnut* RNA with a *K*_d_ of 3.2 μM, while binding to a *boxBA* RNA, which lacked *boxC* and the *boxA-boxC* linker (residues 1–40; Figure 1C), was much weaker (*K*_d_ could not be determined; Figure 5). This observation suggests that binding of SuhB to the *boxA-boxC* linker region and/or *boxC* is important for coordinating the concomitant binding of NusA and NusB/E to *rrnGnut* RNA. This notion is further supported by the observation that SuhB does not facilitate concomitant assembly of NusA and NusB/E on λ/consensus *nut* RNA lacking these regions (Figure 2F). Based on these results, we suggest that SuhB remodels the NusA-*rrnGnut* RNA complex or repositions NusA on *rrnGnut* RNA, by concomitantly interacting with NusA (*via* the AR2 domain) and the RNA (*via* the *boxA-boxC* linker or *boxC*). Such remodeling/repositioning might facilitate subsequent binding of NusB/E.

### Structural basis of the SuhB-NusA^AR2^ interaction

**Figure 6.**
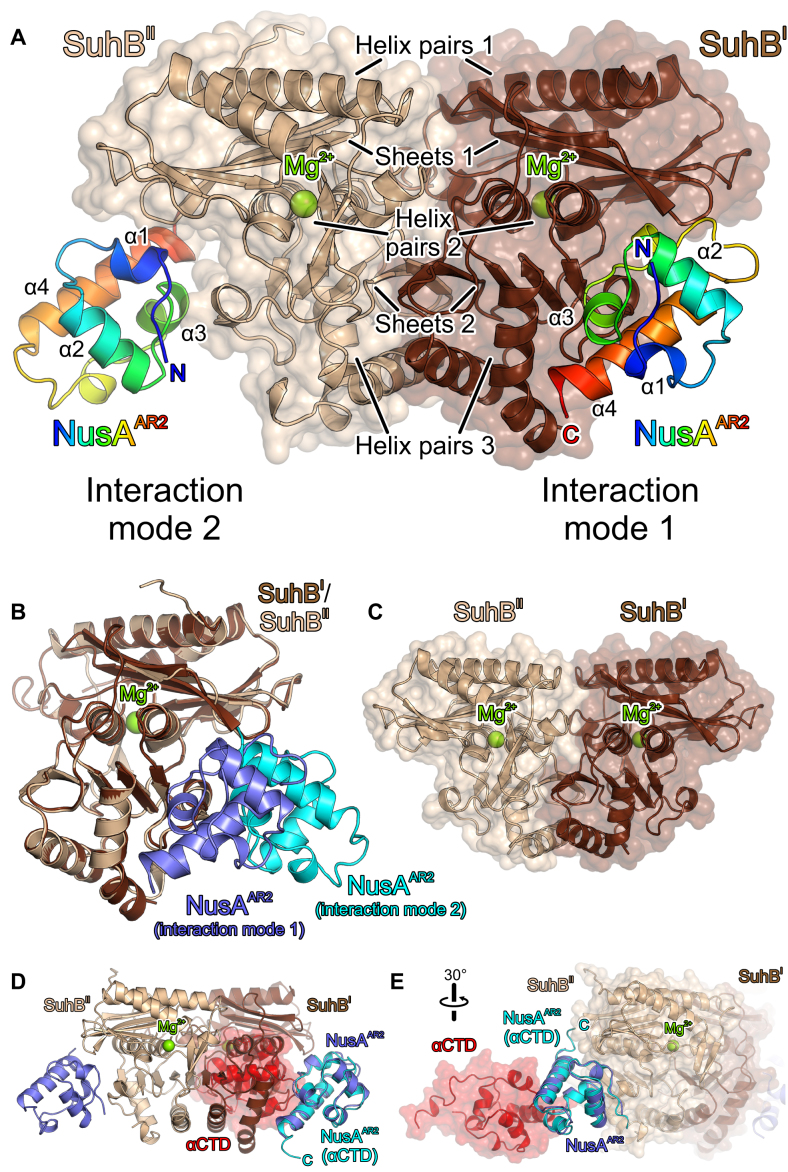
Crystal structures of a SuhB-NusA^AR2^ complex and of isolated wt SuhB. (**A**) SuhB-NusA^AR2^ complex. SuhB – brown and beige; NusA^AR2^ –colored from blue to red (N-terminus to C-terminus). Stacked pairs of helices and sheets as well as bound Mg^2+^ ions are indicated in SuhB, α helices are labeled in NusA^AR2^. An asymmetric unit contains two SuhB molecules (SuhB^I^ and SuhB^II^) and one NusA^AR2^ molecule. However, two symmetry-related NusA^AR2^ molecules are shown to illustrate the different interactions modes with the two SuhB monomers. Interaction modes are identified. (**B**) Superposition of SuhB^II^ (beige) in complex with a molecule of NusA^AR2^ bound *via* interaction mode 2 (cyan) onto SuhB^I^ (brown) in complex with a molecule of NusA^AR2^ bound *via* interaction mode 1 (blue), further illustrating the different binding modes. (**C**) Wt SuhB dimer, shown in the same orientation as the SuhB dimer in (**A**). (**D, E**) αCTD-NusA^AR2^ complex (red and cyan) superimposed *via* the NusA^AR2^ subunit on SuhB-NusA^AR2^ complexes (brown and blue or beige and blue) formed *via* interaction mode 1 (**D**) or interaction mode 2 (**E**). Rotation symbol in (**E**) indicates the view relative to (**A**). Only interaction mode 1 explains the observed mutually exclusive binding of SuhB and αCTD to NusA^AR2^.

To begin elucidating the structural basis underlying the transcriptional functions of SuhB, we determined a crystal structure of a SuhB-NusA^AR2^ complex at 1.65 Å resolution (Table 1; Figure 6A). An asymmetric unit of the crystals contained two SuhB molecules and a single copy of NusA^AR2^. The structures of the two SuhB monomers are almost identical (root-mean-square deviation [rmsd] 0.49 Å for 248 pairs of common Cα atoms; Figure 6B). The SuhB monomers are folded as an alternating stack of three pairs of α helices (helix pairs I–III) and two β sheets (sheets I and II; Figure 6A). The six-stranded, antiparallel sheet I is sandwiched between helix pairs I and II, the five-stranded, mixed sheet II is sandwiched between helix pairs II and III. The AR2 domain formed a dual helix-hairpin-helix motif with four α helices and one 3_10_ helix (order α1-α2-α3–3_10_-α4; Figure 6A), as observed before in other molecular contexts (38,39).

The SuhB monomers formed a near-*C*2-symmetrical dimer (Figure 6A) that closely resembled the previously observed dimer of an isolated SuhB^R184A^ variant (25) (rmsd 0.66 Å for 491 pairs of common Cα atoms). About 1600 Å^2^ of combined surface area are buried at the dimer interface. It has been suggested that wild type (wt) SuhB might form monomers, and that the R184A mutation stabilized the dimer state observed in the crystal structure of SuhB^R184A^ (25). We therefore also crystallized wt SuhB alone and determined its structure at 2.25 Å resolution (Table 1). Isolated wt SuhB crystallized with one monomer in an asymmetric unit, but a dimer essentially identical to the dimer seen with SuhB^R184A^ (rmsd 0.56 Å for 482 pairs of common Cα atoms) and in the SuhB-NusA^AR2^ complex (rmsd 0.49 Å for 497 pairs of common Cα atoms) was formed by crystal symmetry (Figure 6C). Moreover, SEC coupled to multi-angle light scattering confirmed that SuhB forms dimers under the chosen conditions in solution (experimental molecular mass: 60.7 kDa; calculated molecular mass for a SuhB dimer: 58.3 kDa).

**Figure 7.**
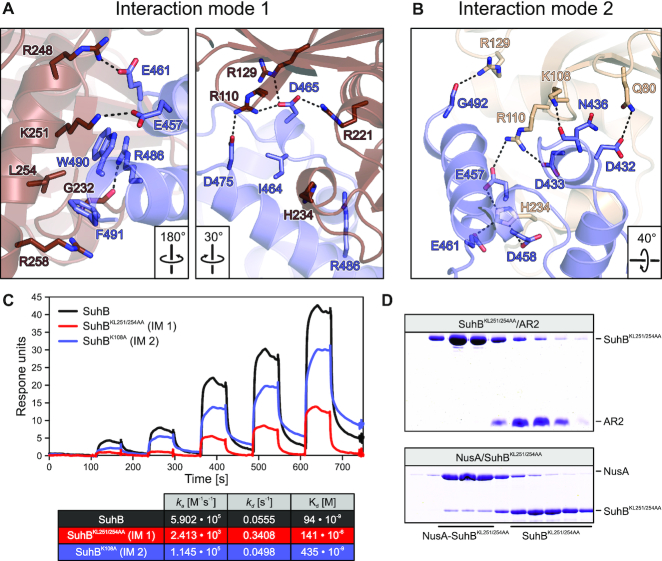
Details of the SuhB-NusA^AR2^ interfaces and mutational analysis. (**A, B**) Details of the interfaces in interaction mode 1 (**A**) and interaction mode 2 (**B**). Interacting residues are shown as sticks and labeled. Atom coloring: carbon – as the respective protein subunit; nitrogen – blue; oxygen – red. Black dashed lines – hydrogen bonds or salt bridges. Rotation symbols indicate the view relative to Figure 6A. (**C**) SPR analyses of SuhB-NusA^AR2^ interactions, employing the indicated SuhB variants. Quantification of the data is provided in the table at the bottom. IM 1/2 – interaction mode 1/2; *k_a_* – association rate constant; *k*_d_ – dissociation rate constant; *K*_d_ – dissociation constant. (**D**) SEC analyses of mixtures of SuhB^KL251/254AA^ (affecting interaction mode 1) with NusA^AR2^ (top) or with NusA^FL^ (bottom). While the interaction with NusA^AR2^ is completely abrogated, it is partially maintained with NusA^FL^.

In the SuhB-NusA^AR2^ crystal, two symmetry-related copies of NusA^AR2^ were bound laterally at equivalent surfaces of the two SuhB subunits of the dimer, i.e. below one exposed edge of sheet I and along the exposed lateral face of helix pair II, sheet II and helix pair III of SuhB (Figure 6A). However, one of the NusA^AR2^ copies associated with one SuhB subunit *via* a flat surface formed by its C-terminal α3–3_10_-α4 portion (interaction mode 1; Figures 6A,B and 7A), while the other AR2 domain bound the second SuhB subunit *via* an edge formed by its α1, α3 and α4 elements (interaction mode 2; Figures 6A, B and 7B).

Interaction modes 1 and 2 bury about 650 Å^2^ and about 500 Å^2^ of combined surface area, respectively, showing that interaction mode 1 encompasses a more extended interface between SuhB and NusA^AR2^. Moreover, superimposing the AR2 domain of a αCTD–NusA^AR2^ complex (38) on the AR2 domains in the SuhB-NusA^AR2^ crystal structure revealed that SuhB bound in interaction mode 1 would preclude αCTD–AR2 interactions (Figure 6D), while interaction mode 2 would allow formation of a ternary SuhB–NusA^AR2^–αCTD complex (Figure 6E). Thus, only interaction mode 1 can explain the mutually exclusive binding of SuhB and αCTD to NusA^AR2^ we observed (Figure 3C,D). We also exchanged interacting residues in the two observed SuhB-AR2 interfaces by site-directed mutagenesis and assessed the effects of these mutations on complex formation between SuhB and NusA^AR2^ by analytical SEC and surface plasmon resonance (SPR) analyses. While all protein variants behaved very similarly to the wt proteins during production and purification, equilibrium circular dichroism (CD) spectra and CD-based thermal melting analyses indicated that at least some of the NusA AR2 domain variants may have suffered changes in 3D structure and/or fold stability. Possibly due to these alterations, SEC analyses did not yield conclusive results. In addition, only wt SuhB, SuhB^KL251/254AA^ (interaction mode 1 affected) and SuhB^K108A^ (interaction mode 2 affected) in combination with wt AR2 gave quantifiable results in SPR runs (Figure 7C). The SPR results confirmed formation of a stable complex between wt SuhB and AR2 with an estimated dissociation constant of *K*_d_ = 94 nM. The analyses were again consistent with interaction mode 1 representing the relevant complex, as SuhB mutations affecting interaction mode 1 led to a reduction of more than three orders of magnitude in the affinity to AR2 (*K*_d_ = 141 μM for SuhB^KL251/254AA^-AR2), while a SuhB mutation affecting interaction mode 2 only had a mild effect (∼4.5-fold reduction in affinity; *K*_d_ = 435 nM for SuhB^K108A^-AR2; Figure 7C). Taken together, the above data suggest that interaction mode 1 represents the SuhB-AR2 interaction mode in solution while interaction mode 2 most likely is a result of crystal packing.

Interestingly, while SuhB^KL251/254AA^ consistently failed to stably bind NusA^AR2^ in SEC as well (Figure 7D, top), a complex with NusA^FL^ remained partially intact during gel filtration (Figure 7D, bottom). Therefore, regions beyond the AR2 domain in NusA^FL^ most likely also contribute to stable complex formation with SuhB.

### SuhB and NusA^AR2^ are required for *rrn* anti-termination

**Figure 8.**
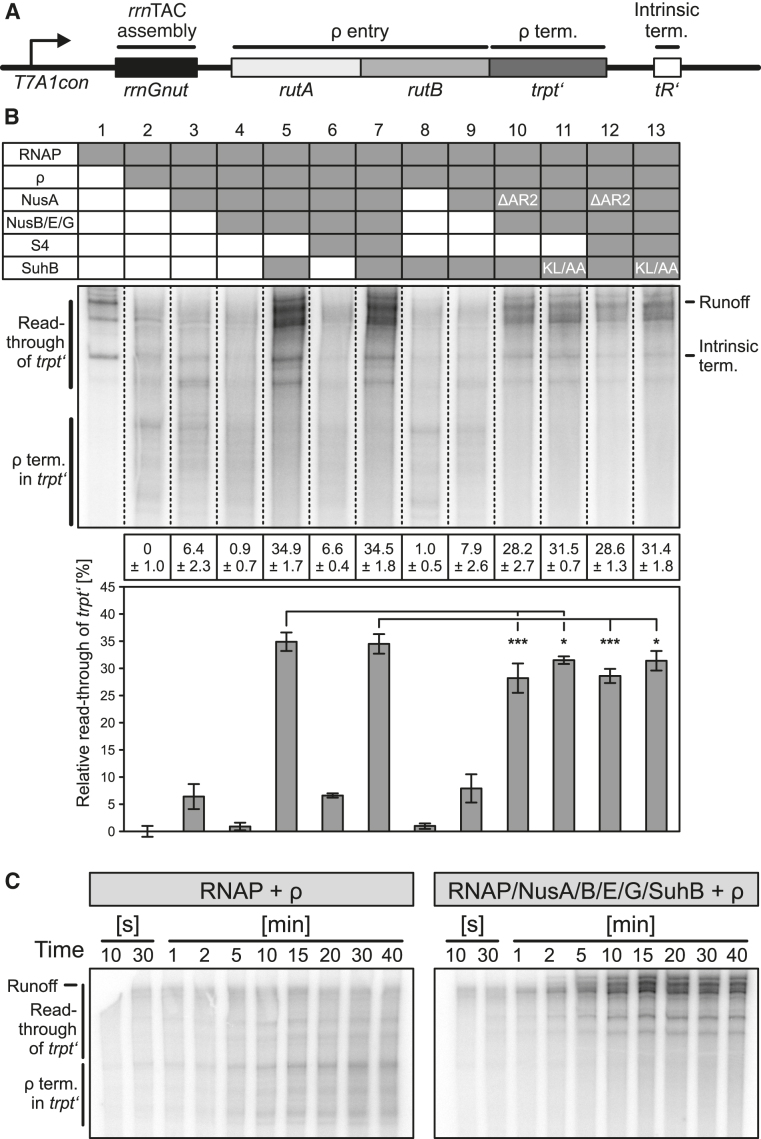
Transcription assays monitoring the effects of SuhB and NusA^AR2^ on ρ-dependent termination. (**A**) Scheme of the DNA employed in the transcription assays. *T7A1con* – promoter; *rutA, rutB* – ρ entry sites; *trpt’* – ρ-dependent terminator; *tR’* – intrinsic terminator. (**B**) Transcription assays monitoring ρ-dependent termination at 15-minute time points by the transcription complexes indicated in the top panel (presence of a component is indicated by a filled box in the table); ΔAR2 – NusA^ΔAR2^; KL/AA – SuhB^KL251/254AA^. Middle panel – samples were analyzed on the same gel, dashed lines are superimposed to facilitate viewing of the figure. RNA products are identified on both sides of the gel; ρ-term. in *trpt’* – transcripts terminating in the *trpt’* region; read-through of *trpt’* – transcripts extending beyond the *trpt’* region. Bottom panels – quantification of the data. Relative read-through of *trpt’* was calculated as the fraction of *trpt’* read-through transcripts relative to all transcripts in a lane, with the value for ρ acting on RNAP alone set to 0 and the values for all other complexes scaled accordingly. Quantified data represent means ± SD of three independent experiments. In this and the following figures, significance was assessed by Student's unpaired *t*-test. Significance indicators in this and the following figures: *P < 0.05; **P < 0.01; ***P < 0.001. (**C**) Long time courses of the same experiments with ρ acting on RNAP alone (left) and on a complex of RNAP, all Nus factors and SuhB (right). Run-off transcripts accumulate in the experiment shown on the right, illustrating anti-ρ activity.

To test the effect of SuhB on transcription, we designed a linear DNA template bearing the *T7A1con* promoter, followed by a region encoding an *rrnGnut* site, *rutA/rutB* ρ entry signals, the *trpt‘* ρ termination region and the *tR’* intrinsic terminator (Figure 8A), and employed this template in *in vitro* transcription assays. Production of an initial, radioactively labeled transcript was initiated at the promoter *via* RNAP holoenzyme by withholding UTP. Subsequently, Nus factors, S4, SuhB and/or ρ were added and transcription was continued by supplying a large excess of all four unlabeled nucleotide tri-phosphates. Samples were taken at 15-minute time points and products were visualized by denaturing PAGE and autoradiography. Products observed under these conditions represent the outcome of single-round transcription events.

As expected, addition of ρ to RNAP alone led to increased termination in the *trpt‘* region (Figure 8B, lanes 1 and 2; note that we quantified relative read-through of the *trpt‘* region). Addition of NusA led to a larger fraction of transcripts extended beyond the *trpt‘* region (Figure 8B, lane 3), consistent with NusA being able to act as a ρ antagonist (10). Further addition of NusG jointly with NusB/E overrode the NusA effect (Figure 8B, lane 4), most likely due to the NusG CTD aiding RNA engagement by ρ (7) (note that we included NusB/E, although results above suggest that in the absence of SuhB the proteins would not efficiently associate). The pattern of ρ-dependent termination upon subsequent addition of S4 resembled the behavior seen with RNAP-NusA (Figure 8B, lane 6), indicating that S4 exhibits some ρ-delaying activity, as noted previously (15). However, only when SuhB was included (with or without S4), ρ-dependent termination was significantly delayed or even suppressed (Figure 8B, lanes 5 and 7). S4 had no significant additional effect in the presence of SuhB (Figure 8B, lanes 5 and 7), but the SuhB effect was clearly dependent on the presence of all Nus factors (Figure 8B, lanes 8 and 9).

Long time courses of the experiment showed that in the presence of SuhB and all Nus factors, transcripts that were elongated beyond the *trpt‘* region accumulated monotonously (Figure 8C). As experiments were performed at high concentrations of ATP and GTP (2 mM and 100 μM, respectively), which are the nucleotides that limit elongation at pause sites, the accumulating transcripts most likely resulted from termination rather than long lived elemental/hairpin-stabilized pausing events. While we cannot fully exclude the possibility that some of these transcripts are the result of prolonged stalling/backtracking of RNAP, SuhB in combination with the Nus factors also led to significant accumulation of run-off transcripts (Figure 8C), confirming that under these conditions SuhB strongly delays and most likely at least partially suppresses ρ-dependent termination.

When we employed NusA^ΔAR2^ instead of NusA^FL^ in the assays, SuhB-mediated delay or suppression of ρ activity was significantly reduced both in the absence (Figure 8B, lane 10) and presence of S4 (Figure 8B, lane 12). Likewise, the SuhB^KL251/254AA^ variant, which exhibits strongly reduced affinity to NusA^AR2^ (Figure 7C), was significantly less efficient than wt SuhB in delaying or suppressing ρ (Figure 8B, lanes 11 and 13). Together, the above findings indicate (i) that SuhB is an essential component of the *rrn*TAC to delay or suppress ρ-dependent termination, (ii) that SuhB requires the Nus factors to elicit its anti-ρ effects and (iii) that a SuhB-NusA^AR2^ interaction contributes to the anti-ρ activity.

**Figure 9.**
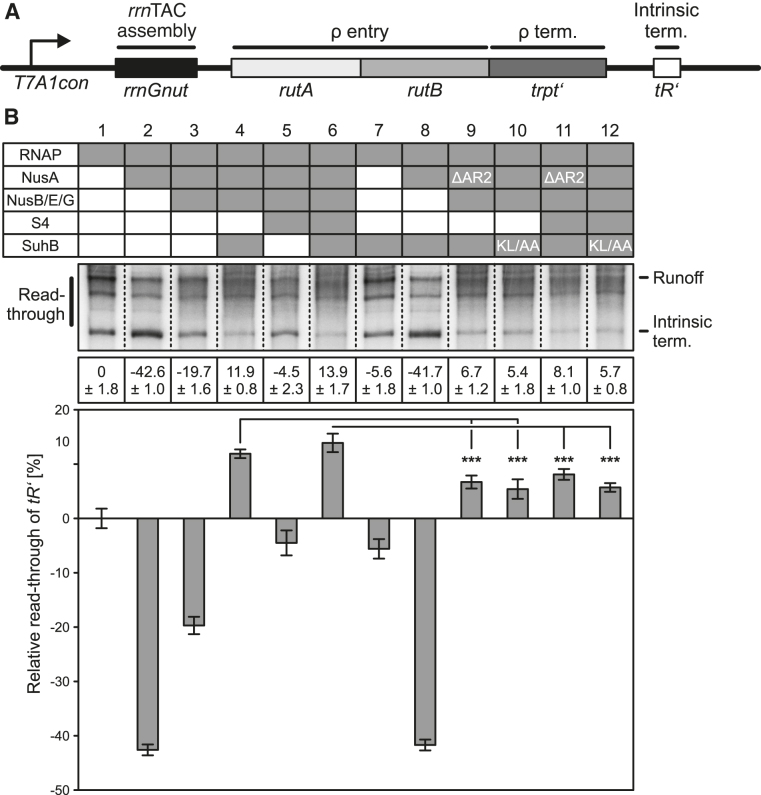
Transcription assays monitoring the effects of SuhB and NusA^AR2^ on intrinsic termination. (**A**) Scheme of the DNA employed in the transcription assays (as in Figure 8A). (**B**) Transcription assays monitoring intrinsic termination efficiency at 15-minute time points by the transcription complexes indicated in the top panel (presence of a component is indicated by a filled box in the table); ΔAR2 – NusA^ΔAR2^; KL/AA – SuhB^KL251/254AA^. Middle panel – samples were analyzed on the same gel, dashed lines are superimposed to facilitate viewing of the figure. RNA products are identified on both sides of the gel; intrinsic term. – transcripts terminating at *tR’*; read-through – transcripts extending beyond *tR’*. Bottom panels – quantification of the data. Relative read-through of *tR’* was calculated as the fraction of *tR’* read-through transcripts relative to all transcripts in a lane, with the value for RNAP alone set to 0 and the values for all other complexes scaled accordingly. Quantified data represent means ± SD of three independent experiments.

We used the same assay to inspect the influence of the factors on intrinsic termination in the absence of ρ (Figure 9A, B). This analysis revealed that intrinsic termination by RNAP was significantly enhanced in the presence of NusA (Figure 9B, lanes 1 and 2; note that we quantified relative read-through of the *tR’* intrinsic terminator), most likely due to the known ability of NusA to stabilize RNA hairpins in the RNA exit tunnel of RNAP (3-6). The NusA effect was reduced stepwise by addition of NusG+NusB/E and NusG+NusB/E+S4 (Figure 9B, lanes 3 and 5). However, only upon addition of SuhB intrinsic termination was significantly suppressed compared to RNAP alone (Figure 9B, lanes 4 and 6). Again, the SuhB effect was largely independent of S4 (Figure 9B, lanes 4 and 6) but strongly dependent on the presence of all Nus factors (Figure 9B lanes 7 and 8). As for ρ-dependent termination, suppression of intrinsic termination by SuhB depended in part on the presence of a NusA AR2 domain (Figure 9B, lanes 9 and 11) and on an intact binding site on SuhB for NusA^AR2^ (Figure 9B, lanes 10 and 12). Thus, in the context of all Nus factors, SuhB is able to override the intrinsic termination-supporting function of NusA, most likely in part due to an interaction with the NusA AR2 domain.

## DISCUSSION

The structural details underlying formation of a transcription complex that is specialized for rRNA synthesis in *E. coli*, the *rrn*TAC that involves RNAP, a *nut*-like site on the transcript, NusA, B, E and G, r-protein S4 and SuhB, are presently not known. The *nut*-like signal element renders the *rrn*TAC specific for transcription of rRNA operons. As an initial step to characterize this complex on the molecular level, we have investigated the interactions of the SuhB subunit with other *rrn*TAC components and the functional consequences of the detected interactions for the assembly of a *rrn*TAC and for the ability of the *rrn*TAC to counteract ρ-dependent and intrinsic transcription termination.

### Inter-dependencies of factors in the assembly and function of an *rrn*TAC

We find that SuhB stably interacts with the AR2 domain of NusA (Figures 3A and 7C) and determined a crystal structure of a SuhB-NusA^AR2^ complex (Figure 6A). However, in the presence of *rrnGnut* RNA, the SuhB-NusA^AR2^ interaction is dispensable for stable integration of SuhB into an *rrn*TAC (Figure 4C). Our results suggest that interaction of an RNAP αCTD with NusA^AR2^ might sequester the AR2 domain from SuhB in the absence of *rrnGnut* RNA and lead to failure of SuhB to integrate into such complexes. By engaging in interactions with multiple components of the *rrn*TAC, *rrnGnut* RNA may weaken the αCTD-AR2 interaction. Additionally, *rrnGnut* RNA may increase the local concentration of SuhB so that SuhB can compete with αCTD for AR2 binding. Irrespective of the precise mechanism, our data strongly support the view that a SuhB-AR2 interaction ensues in the assembled *rrn*TAC, as deletion of AR2 from NusA or disruption of the SuhB-AR2 interaction interfered with SuhB-dependent ρ and intrinsic anti-termination (Figures 8B and 9B).

ChIP analyses using reporter systems that lacked NusB or *boxA* (16) and the weakness of the reported SuhB–RNAP holoenzyme interaction (25) suggested that SuhB is incorporated into the *rrn*TAC in a NusB/*boxA*-dependent manner (16). However, SuhB deletion also led to a depletion of the NusB ChIP signal (16), leaving open the question of which factor is responsible for stable incorporation of the other. Our results clearly support a functional interplay between SuhB, NusA, the NusB/E dimer and *rrnGnut* RNA in *rrn*TAC assembly, and they suggest that SuhB is required for subsequent integration of NusB/E rather than *vice versa*. Binding of NusA to *rrnGnut* RNA was incompatible with concomitant binding of NusB/E, but NusB/E binding was possible when SuhB was additionally present (Figure 2E). NusA might interfere with NusB/E binding by sterically blocking NusB/E or by occupying the *boxA* element, which is the known interaction region of NusB/E (20,21,40,41). SuhB-NusA contacts may lead to a different conformation of NusA on *rrnGnut* RNA that no longer blocks NusB/E access to *boxA*. Alternatively, SuhB, based on its affinity to the *boxA-boxC*-linker/*boxC* region of *rrnGnut* RNA, may guide NusA to this part of the RNA, granting NusB/E access to *boxA*. Together with a previous crystal structure showing that a S1-KH1-KH2 fragment of *M. tuberculosis* NusA can bind a *boxC*-like RNA element (22), our findings are consistent with NusB/E binding to *boxA*, SuhB to the *boxA*-*boxC* linker and NusA to *boxC* in the final complex.

Most likely, additional interactions of SuhB with other components of an *rrn*TAC, including RNAP itself (25), ensue in the fully assembled complex. It has been shown that other transcription elongation and anti-termination complexes assemble cooperatively based on many binary interactions among the participating factors, which are not necessarily all stable in isolation (19). Indeed, our interaction studies provided evidence that NusA^FL^ establishes at least one additional contact to SuhB beyond the AR2 domain (Figure 7D). Moreover, our transcription assays showed that other Nus factors are also required for SuhB to unfold its negative effects on ρ-dependent and intrinsic termination (Figures 8B and 9B), supporting the idea that more complex, multi-factorial interactions ensue around SuhB in the complete *rrn*TAC. Finally, our finding that stable binding of S4 to an *rrn*TAC partly depends on SuhB (Figure 4G) could be due to direct SuhB-S4 interactions in the full complex.

### Molecular basis of transcription anti-termination by an *rrn*TAC

It has been proposed that the main function of the *rrn*TAC does not lie in the suppression of premature transcription termination but rather in its support for co-transcriptional rRNA folding, maturation and ribosomal subunit assembly (16,42). However, these conclusions were based on studies with individual *nusB* or *suhB* knockout strains, in which residual anti-termination activity based on the other factors may still remain. Our results clearly demonstrate that a transcription complex minimally comprising *rrnGnut* RNA, all Nus factors and SuhB efficiently counteracts or delays ρ-dependent termination and likewise interferes with intrinsic termination.

We showed that SuhB is responsible to a large part for the ability of an *rrn*TAC to subdue ρ-dependent as well as intrinsic termination. Deletion of the NusA AR2 domain or disruption of the SuhB–NusA^AR2^ interaction clearly reduced both types of anti-termination activities (Figures 8B and 9B). A recent cryo-electron microscopic structure of a NusA-stabilized *his* operon hairpin-paused transcription complex suggested that an observed αCTD-NusA^AR2^ contact may contribute to the positioning of NusA on RNAP in a pause hairpin-stabilizing conformation (6). A similar conformation of NusA on RNAP can be expected when it exerts its role in supporting intrinsic termination. Thus, SuhB binding the NusA AR2 domain, which as we showed is mutually exclusive with the αCTD-NusA^AR2^ interaction (Figure 3C, D), may contribute to a different positioning of NusA on RNAP, such that its ability to stabilize RNA hairpins in the RNA exit tunnel of RNAP is prevented or reduced. We recently showed that a similar principle is at work during λN-dependent processive anti-termination, mediated by the λN protein (18,19).

How SuhB contributes to anti-ρ activity is presently unclear, but it may again involve similar principles as suggested for λN-mediated ρ anti-termination. In the structure of a λN-TAC (18,19), the NusG CTD, which aids ρ in engaging *rut* sites (7), is sequestered by alternative interactions with NusA and NusE. However, additional mechanisms contribute to counteracting ρ in the λN-TAC, possibly involving steric hindrance that would prevent ρ from approaching its binding site(s) on RNAP (18,19). Likewise, SuhB in conjunction with the Nus factors might prevent ρ from productively contacting RNAP, as is required for ρ-dependent termination (43).

### Implications for co-transcriptional rRNA folding, processing and ribosome assembly

A ‘delivery’ model has been proposed to explain the role of the *rrn*TAC in co-transcriptional folding and maturation of rRNA (13,42,44). In this model, the RNAP-modifying components of the *rrn*TAC hold on to the RNA *nut*-like element encoded in the leader and spacer regions of the *rrn* operons, thereby keeping following regions of the transcript in close proximity of the RNAP RNA exit tunnel. When 3′-regions of rRNA domains are synthesized at later stages of transcription and emerge from the exit tunnel, they would thus be presented with 5′-regions that they need to pair with. The findings that NusB and *boxA* constitute crucial components for the functioning of the *rrn*TAC (14,16,42) are consistent with this idea, as the NusB/E dimer establishes contacts to *boxA*. Our data suggest that there might be a more elaborate multi-protein–RNA interaction on the *rrn*TAC that mediates the delivery mechanism or that contributes in other ways to rRNA maturation, as SuhB and NusA apparently reinforce the grip of the *rrn*TAC on the transcript leader/spacer region by contacts to the *nut*-like RNA element (Figure 5), and as SuhB is required for efficient incorporation of the NusB/E dimer in the presence of NusA (Figure 4G, H).

## DATA AVAILABILITY

Structure factors and coordinates have been deposited in the RCSB Protein Data Bank (https://www.rcsb.org/) with accession codes 6IB7 (SuhB) and 6IB8 (SuhB-NusA^AR2^).
